# Supplementary material for: Early biological markers of post-acute sequelae of SARS-CoV-2 infection
Source: Nat Commun. 2024 Aug 29;15:7466. doi: 10.1038/s41467-024-51893-7 (PMC11358427; doi:10.1038/s41467-024-51893-7)
Supplement: Supplementary file 1 — Supplementary Information [file 41467_2024_51893_MOESM1_ESM.pdf]

**Early Biological Markers of Post-Acute Sequelae of SARS-CoV-2 Infection**

**Supplemental Table 1:** Participant characteristics by outcome status.

| | Non-PASC | PASC | Total |
|---|---|---|---|
| | N=72 | N=32 | N=104 |
| Age, years (median [IQR]) | 32 (22.5 to 42) | 39 (31.5 to 47.5) | 35.5 (27 to 43.5) |
| Sex, n (%) | | | |
| Female | 36 (50%) | 17 (53%) | 53 (51%) |
| Male | 36 (50%) | 15 (47%) | 51 (49%) |
| Race/Ethnicity, n (%) | | | |
| Hispanic or Latino Ethnicity | 14 (19%) | 6 (19%) | 20 (19%) |
| White | 40 (56%) | 19 (59%) | 59 (57%) |
| Black or African American | 3 (4%) | 1 (3%) | 4 (4%) |
| Asian | 10 (14%) | 4 (13%) | 14 (13%) |
| Pacific Islander/Native Hawaiian | 1 (1%) | 1 (3%) | 2 (2%) |
| American Indian or Alaska Native | 1 (1%) | 1 (3%) | 2 (2%) |
| Prefer not to answer | 3 (5%) | 0 (0%) | 3 (3%) |
| Vaccination status, n (%) | | | |
| Unvaccinated | 47 (64%) | 21 (68%) | 68 (65%) |
| Fully vaccinated[a] | 26 (36%) | 10 (32%) | 36 (35%) |
| BMI, n (%) | | | |
| <25 $kg/m^2$ | 39 (58%) | 12 (35%) | 51 (51%) |
| 25 to 29.9 | 20 (29%) | 7 (23%) | 27 (27%) |
| >30 | 9 (13%) | 13 (42%) | 22 (22%) |
| Education, n (%) | | | |
| Less than HS | 4 (7%) | 2 (7%) | 6 (7%) |
| At least some HS | 6 (11%) | 3 (10%) | 9 (11%) |
| At least some college | 29 (53%) | 13 (43%) | 42 (49%) |
| At least some graduate school | 16 (29%) | 12 (40%) | 28 (33%) |
| Variant[b], n (%) | | | |
| Pre-Delta | 41 (56%) | 20 (65%) | 61 (59%) |
| Delta | 26 (36%) | 10 (32%) | 36 (35%) |
| Omicron | 6 (8%) | 1 (3%) | 7 (7%) |
| Any comorbidity[c], n (%) | | | |
| No | 58 (79%) | 22 (71%) | 80 (77%) |
| Yes | 15 (21%) | 9 (29%) | 24 (23%) |
| Number of symptoms in acute illness | 7 (3 to 12) | 12 (9 to 14) | 9 (4 to 13) |

Abbreviations: BMI: body mass index; HS: high school.

[a]Fully vaccinated was defined as completion of primary vaccination series with 2 week window prior to symptom onset.

[b]Variant status was defined by viral sequencing result for 70 (67%) participants and by calendar time for the remaining 34 (33%) participants.

[c]Any comorbidity was defined from the following list: history of autoimmune disease, cancer in the past 2 years, diabetes, heart disease, hypertension, lung disease, or kidney disease

**Supplemental Table 2:** Participant characteristics by outcome status, restricted to those who received a blood draw.

| | No PASC | PASC | Total |
|---|---|---|---|
| | N=52 | N=28 | N=80 |
| Age, years (median [IQR]) | 37 (29-44) | 39.5 (32.5-48.5) | 38 (30-45) |
| Sex, n (%) | | | |
| Female | 28 (54) | 13 (46) | 41 (51) |
| Male | 24 (46) | 15 (54) | 39 (49) |
| Race/Ethnicity, n (%) | | | |
| Hispanic or Latino Ethnicity | 10 (20) | 5 (18) | 15 (19) |
| White | 27 (54) | 17 (61) | 44 (56) |
| Black or African American | 2 (4) | 1 (4) | 3 (4) |
| Asian | 9 (18) | 3 (11) | 12 (15) |
| Prefer not to answer | 2 (4) | 2 (7) | 4 (5) |
| Vaccination status, n (%) | | | |
| Unvaccinated | 30 (58) | 20 (71) | 50 (63) |
| Fully vaccinated[a] | 22 (42) | 8 (29) | 30 (38) |
| BMI, n (%) | | | |
| <25 | 23 (47) | 11 (39) | 34 (44) |
| 25 to 29.9 | 18 (37) | 6 (21) | 24 (31) |
| >30 | 8 (16) | 11 (39) | 19 (25) |
| Education, n (%) | | | |
| Less than HS | 3 (6) | 2 (7) | 5 (6) |
| At least some HS | 6 (12) | 3 (11) | 9 (12) |
| At least some college | 27 (53) | 11 (41) | 38 (49) |
| At least some graduate school | 15 (29) | 11 (41) | 26 (33) |
| Variant[b], n (%) | | | |
| Pre-Delta | 31 (60) | 19 (68) | 50 (63) |
| Delta | 21 (40) | 9 (32) | 30 (38) |
| Omicron | 0 (0) | 0 (0) | 0 (0) |
| Any comorbidity[c], n (%) | | | |
| No | 38 (73) | 20 (71) | 58 (73) |
| Yes | 14 (27) | 8 (29) | 22 (28) |
| Number of symptoms in acute illness | 9 (4.5 to 14) | 12 (10 to 14.5) | 11 (6 to 14) |

Abbreviations: BMI: body mass index; HS: high school.

[a]Fully vaccinated was defined as completion of primary vaccination series with 2-week window prior to symptom onset.
[b]Variant status was defined by viral sequencing result for 70 (67%) participants and by calendar time for the remaining 34 (33%) participants.
[c]Any comorbidity was defined from the following list: history of autoimmune disease, cancer in the past 2 years, diabetes, heart disease, hypertension, lung disease, or kidney disease

**Supplemental Table 3: Prevalence of reported symptoms among those with PASC.**

| PASC Symptom | Prevalence, n (%) |
|---|---|
| Chills | 3 (9%) |
| Fatigue | 8 (25%) |
| Cough | 3 (9%) |
| Shortness of breath | 6 (19%) |
| Chest pain | 2 (6%) |
| Palpitations | 5 (16%) |
| Rhinorrhea | 8 (25%) |
| Sore throat | 4 (13%) |
| Muscle pain/weakness | 1 (3%) |
| Loss of appetite | 1 (3%) |
| Nausea | 1 (3%) |
| Constipation | 3 (9%) |
| Diarrhea | 7 (22%) |
| Rash | 6 (19%) |
| Trouble with taste/smell | 1 (3%) |
| Phantosmia | 1 (3%) |
| Trouble with concentration/memory | 14 (44%) |
| Headache | 6 (19%) |
| Trouble with vision | 3 (9%) |
| Dizziness | 6 (19%) |
| Trouble with balance | 1 (3%) |
| Paresthesia | 3 (9%) |
| Joint pain | 4 (13%) |
| Back pain | 7 (22%) |
| Trouble with sleep | 9 (28%) |
| Menstrual cramps | 2 (6%) |

**Supplementary Table 4.** Association of virologic factors with post-acute sequelae of SARS-CoV-2 infection (PASC) with minimized false discovery calculation (Q-value).

| | Non-PASC N=73, median (IQR) | PASC N=31, median (IQR) | Incidence Ratios[c] (95% CI) | p-value | Q-value |
|---|---|---|---|---|---|
| Maximum RNA N viral load[a] | 6.7 (4.8 to 8.3) | 8.3 (7.2 to 9.5) | 1.21 (1.02 to 1.43) | p = 0.02 | q = 0.08 |
| Maximum RNA E viral load | 6.3 (4.7 to 7.9) | 7.6 (6.7 to 8.6) | 1.18 (1.01 to 1.39) | p = 0.03 | q = 0.08 |
| Maximum infectious viral load[a,b] | 5.3 (3.0 to 6.1) | 5.2 (4.2 to 5.9) | 1.08 (0.90 to 1.32) | p = 0.43 | q = 0.43 |
| Rate of RNA N viral decay | -0.3 (-0.5 to -0.1) | -0.4 (-0.5 to -0.2) | 0.66 (0.26 to 1.70) | p = 0.39 | q = 0.43 |
| Rate of RNA E viral decay | -0.4 (-0.6 to -0.2) | -0.5 (-0.7 to -0.3) | 0.73 (0.46 to 1.16) | p = 0.19 | q = 0.23 |

[a]Viral load measurements were log-transformed copies/mL.

[b]Among 104 participants with a viral measurement, there were 94 (90%) who had maximum infectious viral load assessed.

[c]Incidence rate ratios calculated with delta-method standard errors.

We employed Storey Q-values in evaluating virologic factors measured by GEE to strike a balance between controlling errors and maximizing the ability to detect true associations. The base data is derived from two-sided hypothesis testing. We found similar results with and without correction for multiple hypothesis testing. Interpretation of these results can be further elaborated considering q-values in the context of Bayesian posterior probabilities. There remains debate over the use of Storey Q-values and other techniques for multiple corrections in clinical research. We interpret a q-value of 0.083 as consistent with an association between higher maximum viral load and PASC as a correct discovery with probability of 91.7% (1 – 0.083).

**Supplemental Figure 1.** Non-imputed inflammatory marker levels among those with and without post-acute sequelae of SARS-CoV-2 infection (PASC) over a 28-day period after symptom onset.

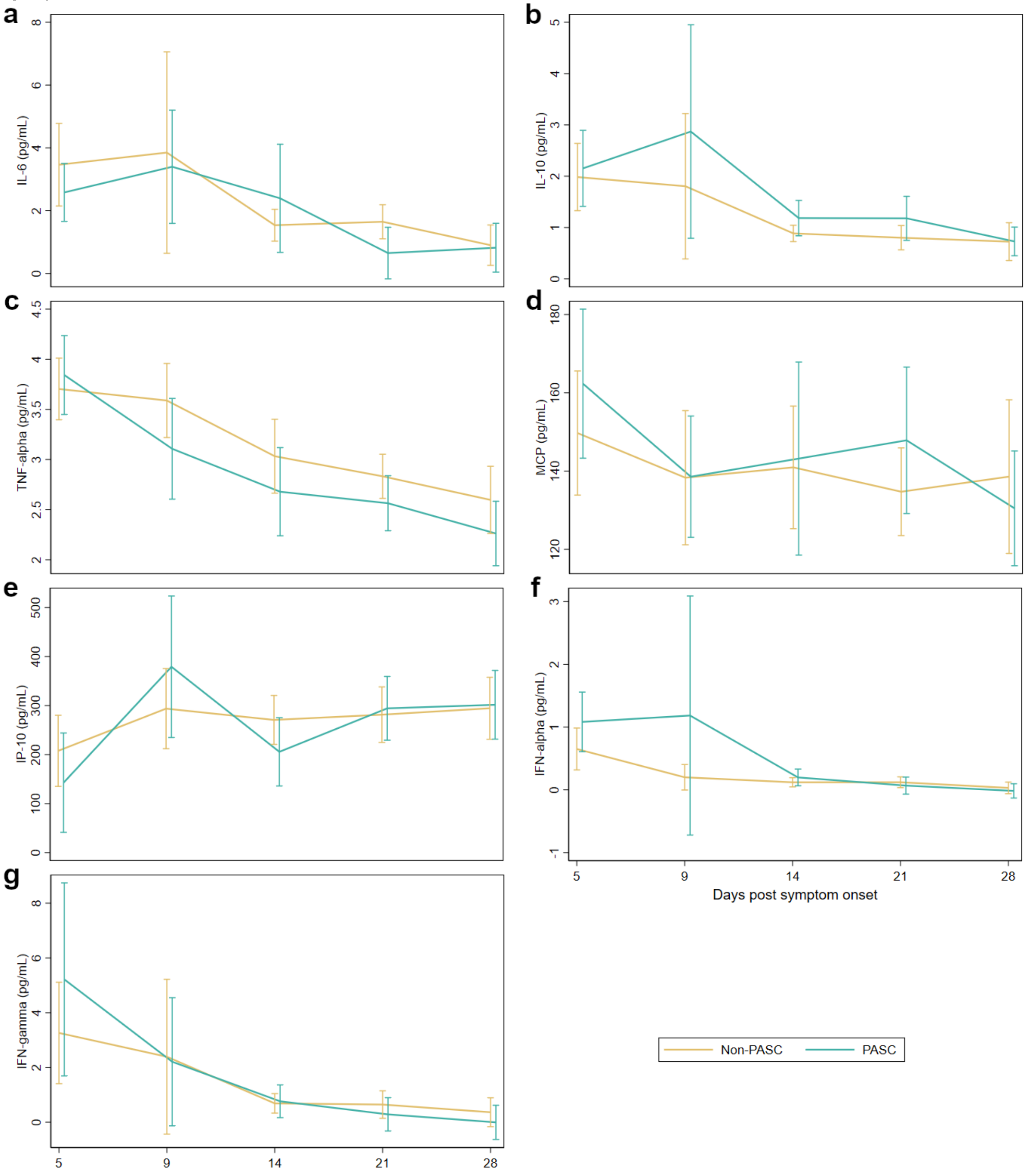


**a-g:** Estimated mean values and 95% confidence intervals are plotted for biological specimen among PASC and non-PASC groups at days 5, 9, 14, 21 and 28 for (**a**) IL-6, (**b**) IL-10, (**c**) TNF-alpha, (**d**) MCP, (**e**) IP-10, (**f**) IFN-alpha, and (**g**) IFN-gamma using generalized estimating equations fit with independent correlation, identity linkage, and Gaussian distribution. Covariates included sex, age, vaccination status, and SARS-CoV-2 variant. All statistics are derived from multiply imputed data from 80 IDs. Statistical significance was assessed using two-tailed tests. No

statistically significant differences in estimated values were found when comparing PASC vs. non-PASC at each time point. Source data are provided as a Source Data file.

**Supplementary Table 5.** Oligonuceotide sequences for RT-PCR

| | Forward primer | Probe | Reverse primer |
|---|---|---|---|
| SARS-CoV-2 N | GACCCCAAAATCAGCGAAAT | ACCCCGCATTACGTTTGGTGGACC | TCTGGTTACTGCCAGTTGAATCTG |
| SARS-CoV-2 E | ACAGGTACGTTAATAGTTAATAGCGT | ACACTAGCCATCCTTACTGCGCTTCG | ATATTGCAGCAGTACGCACACA |
| RNaseP | AGATTTGGACCTGCGAGCG | TTCTGACCTGAAGGCTCTGCGCG | CGGCTGTCTCCACAAGT |
