# Supplementary material for: Early biological markers of post-acute sequelae of SARS-CoV-2 infection
Source: Nat Commun. 2024 Aug 29;15:7466. doi: 10.1038/s41467-024-51893-7 (PMC11358427; doi:10.1038/s41467-024-51893-7)
Supplement: Supplementary file 3 — Reporting Summary [file 41467_2024_51893_MOESM3_ESM.pdf]

nature portfolio

Corresponding author(s): Scott Lu

Last updated by author(s): 01-Aug-2024

Reporting Summary

Nature Portfolio wishes to improve the reproducibility of the work that we publish. This form provides structure for consistency and transparency in reporting. For further information on Nature Portfolio policies, see our Editorial Policies and the Editorial Policy Checklist.

Statistics

For all statistical analyses, confirm that the following items are present in the figure legend, table legend, main text, or Methods section.

n/a	Confirmed	
☐	☒	The exact sample size (*n*) for each experimental group/condition, given as a discrete number and unit of measurement
☐	☒	A statement on whether measurements were taken from distinct samples or whether the same sample was measured repeatedly
☐	☒	The statistical test(s) used AND whether they are one- or two-sided<br>*Only common tests should be described solely by name; describe more complex techniques in the Methods section.*
☐	☒	A description of all covariates tested
☐	☒	A description of any assumptions or corrections, such as tests of normality and adjustment for multiple comparisons
☐	☒	A full description of the statistical parameters including central tendency (e.g. means) or other basic estimates (e.g. regression coefficient) AND variation (e.g. standard deviation) or associated estimates of uncertainty (e.g. confidence intervals)
☐	☒	For null hypothesis testing, the test statistic (e.g. *F*, *t*, *r*) with confidence intervals, effect sizes, degrees of freedom and *P* value noted<br>*Give P values as exact values whenever suitable.*
☐	☒	For Bayesian analysis, information on the choice of priors and Markov chain Monte Carlo settings
☐	☒	For hierarchical and complex designs, identification of the appropriate level for tests and full reporting of outcomes
☒	☐	Estimates of effect sizes (e.g. Cohen's *d*, Pearson's *r*), indicating how they were calculated

Our web collection on statistics for biologists contains articles on many of the points above.

Software and code

Policy information about availability of computer code

Data collection	Study data were collected and managed in REDCap (Research Electronic Data Capture) electronic data capture tools hosted by UCSF. REDCap is a secure, web-based software platform designed to support data capture for research studies, providing 1) an intuitive interface for validated data capture; 2) audit trails for tracking data manipulation and export procedures; 3) automated export procedures for seamless data downloads to common statistical packages; and 4) procedures for data integration and interoperability with external sources.
Data analysis	Data was analyzed using Stata SE v16.1 and R package 'qvalue'

For manuscripts utilizing custom algorithms or software that are central to the research but not yet described in published literature, software must be made available to editors and reviewers. We strongly encourage code deposition in a community repository (e.g. GitHub). See the Nature Portfolio guidelines for submitting code & software for further information.

Data

Policy information about availability of data

All manuscripts must include a data availability statement. This statement should provide the following information, where applicable:

- Accession codes, unique identifiers, or web links for publicly available datasets
- A description of any restrictions on data availability
- For clinical datasets or third party data, please ensure that the statement adheres to our policy

Data that support the findings of this study have been deposited in https://figshare.com/s/b9c66098e3fa3779deaf (https://doi.org/10.6084/m9.figshare.26346589).

Research involving human participants, their data, or biological material

Policy information about studies with human participants or human data. See also policy information about sex, gender (identity/presentation), and sexual orientation and race, ethnicity and racism.

Reporting on sex and gender	This study collected both sex assigned at birth and gender identity. The current analysis uses sex assigned at birth which was found to be in agreement with gender identity.
Reporting on race, ethnicity, or other socially relevant groupings	We queried participants on whether they identified as Hispanic/Latino in ethnicity and then which race they identified as from the following: 1) American native or Alaskan native; 2) Asian; 3) Black or African American; 4) Native Hawaiian or Other Pacific Islander; 5) White; or Prefer not to answer. We used a combined race/ethnicity variable to indicate Hispanic/Latino Caucasian/White from non-Hispanic/Latino Caucasian/White categories.
Population characteristics	Participants ranged in age from 1 to 78, and included unvaccinated and vaccinated participants; pre-Delta, Delta, and Omicron SARS-CoV-2 strains; symptomatic and asymptomatic
Recruitment	Potential participants were identified based on a daily report of positive SARS-CoV-2 PCR testing at a UCSF-affiliated laboratory. Research staff called potential participants within 5 days of their illness onset to determine eligibility and offer enrollment to the study. Potential for selection bias exists in participant retention in the post-acute follow-up period; participants who were more affected by COVID-19 could have greater motivation to follow-up, which could result in a greater retention among those who develop PASC.
Ethics oversight	The study was reviewed by the University of California, San Francisco (UCSF) Institutional Review Board and given a designation of public health surveillance according to federal regulations as summarized in 45 CFR 46.102(d)(1)(2).

Note that full information on the approval of the study protocol must also be provided in the manuscript.

Field-specific reporting

Please select the one below that is the best fit for your research. If you are not sure, read the appropriate sections before making your selection.

- [x] Life sciences
- [ ] Behavioural & social sciences
- [ ] Ecological, evolutionary & environmental sciences

For a reference copy of the document with all sections, see nature.com/documents/nr-reporting-summary-flat.pdf

Life sciences study design

All studies must disclose on these points even when the disclosure is negative.

Sample size	All eligible study participants were included in this analysis.
Data exclusions	No data were excluded from these analyses.
Replication	Results were replicated by study analysts using the same source data.
Randomization	Participants were classified into PASC and non-PASC groups based on their symptom status at their post-acute study visit.
Blinding	Blinding was not possible during data collection as the collected data directly translates to group allocation.

Reporting for specific materials, systems and methods

We require information from authors about some types of materials, experimental systems and methods used in many studies. Here, indicate whether each material, system or method listed is relevant to your study. If you are not sure if a list item applies to your research, read the appropriate section before selecting a response.

Materials & experimental systems

n/a	Involved in the study	
x		Antibodies
x		Eukaryotic cell lines
x		Palaeontology and archaeology
x		Animals and other organisms
x		Clinical data
x		Dual use research of concern
x		Plants

Methods

n/a	Involved in the study	
x		ChIP-seq
x		Flow cytometry
x		MRI-based neuroimaging

Plants

Seed stocks	*Report on the source of all seed stocks or other plant material used. If applicable, state the seed stock centre and catalogue number. If plant specimens were collected from the field, describe the collection location, date and sampling procedures.*
Novel plant genotypes	*Describe the methods by which all novel plant genotypes were produced. This includes those generated by transgenic approaches, gene editing, chemical/radiation-based mutagenesis and hybridization. For transgenic lines, describe the transformation method, the number of independent lines analyzed and the generation upon which experiments were performed. For gene-edited lines, describe the editor used, the endogenous sequence targeted for editing, the targeting guide RNA sequence (if applicable) and how the editor was applied.*
Authentication	*Describe any authentication procedures for each seed stock used or novel genotype generated. Describe any experiments used to assess the effect of a mutation and, where applicable, how potential secondary effects (e.g. second site T-DNA insertions, mosiacism, off-target gene editing) were examined.*
